# A Case of Low-Flow Priapism as a Complication of COVID-19 Infection

**DOI:** 10.7759/cureus.22613

**Published:** 2022-02-25

**Authors:** Saad Ahmed, Ouiam Akotat, Varsha Sajeesh, Mutallab Alabi, Soumendra Datta

**Affiliations:** 1 Internal Medicine, Colchester Hospital, East Suffolk and North Essex National Health Service (NHS) Foundation Trust, Colchester, GBR; 2 Urology, Colchester Hospital, East Suffolk and North Essex National Health Service (NHS) Foundation Trust, Colchester, GBR

**Keywords:** disseminated intravascular coagulation (dic), vasculopathy, extra-pulmonary manifestation, covid-19, low flow priapism

## Abstract

On 11 March 2020, the World Health Organisation (WHO) declared the severe acute respiratory syndrome coronavirus 2 (SARS-COV-2) (COVID-19) a pandemic. With a global incidence of over 414 million cases, as of 16 February 2022, it presents a significant burden on healthcare. COVID-19 is primarily considered a respiratory illness; however, a wide range of presentations have been reported including a tendency for thrombotic complications. We report a case of a 58-year-old man who presented with dyspnoea, pyrexia and dry cough. Upon admission, he was noted to be in a severe type 1 respiratory failure with bilateral pulmonary infiltrates suggestive of COVID-19 infection. Rapid transfer to intensive therapy unit (ITU) ensued with intubation and ventilation. The patient was noted to have developed priapism one day following admission with subsequent aspiration by the Urology team, achieving detumescence. Priapism is a state of persistent penile erection that continues for four hours beyond sexual stimulation. Our case highlights the role of thrombosis, dysregulation of the clotting cascade and acute disseminated intravascular coagulation (DIC) as shared pathologies in priapism and COVID-19 infection. We put forth an example of one of the extra-pulmonary manifestations of the COVID-19 secondary to the pro-thrombotic state associated with the COVID-19 infection.

## Introduction

On 11 March 2020, the World Health Organisation (WHO) declared the severe acute respiratory syndrome coronavirus 2 (SARS-COV-2) (COVID-19) a pandemic [1]. COVID-19 is primarily considered a respiratory illness; however, a wide range of presentations have been reported including a tendency for thrombotic complications. In this paper, we present a case of priapism in a COVID-19-positive patient secondary to the pro-thrombotic state augmented by this illness.

## Case presentation

**Figure 1:**
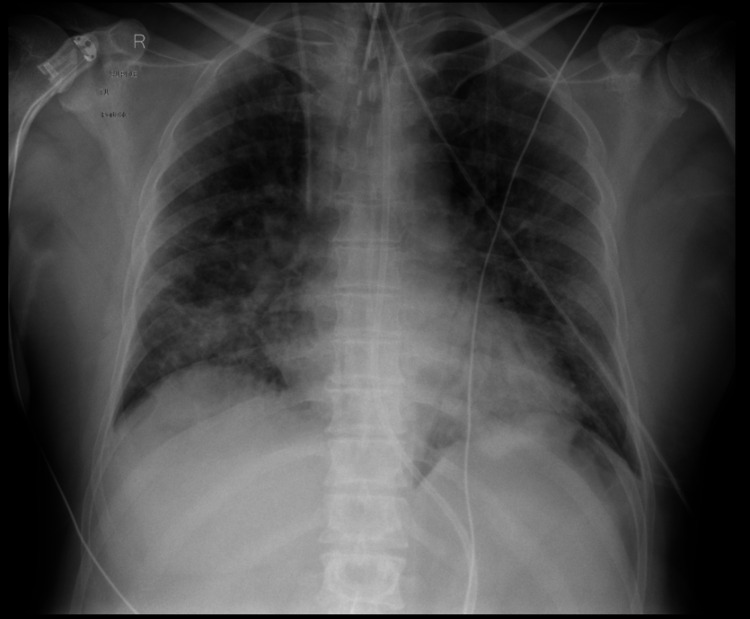
Chest radiograph Chest radiograph showing extensive bilateral pulmonary nodular infiltrates.

A 58-year-old male lorry driver of Turkish descent presented with progressively worsening shortness of breath, fever and cough. A prodromal flu-like illness lasting eight days preceded admission as an inpatient in our hospital with rigours and intermittent temperature spikes. The patient was self-isolating at home with his wife but attended the hospital due to shortness of breath and respiratory distress, which started suddenly 24 hours prior and gradually worsened. There was no recent travel history. Co-morbidities included hypertension that was well controlled with amlodipine monotherapy. The patient quit smoking 30 years prior to admission and had no family history of urological disease or malignancy, no history of psychoactive drug use or any other drug abuse. The patient reported no prior urological symptoms. Urine dipstick test and urine toxicology screen were negative. Initial observations included severe hypoxia with oxygen saturation of 88% on 15 litres of oxygen delivered via a non-rebreather mask, heart rate of 110 beats/minute, blood pressure of 110/70 mmHg and temperature of 38.5°C. Initial blood tests are summarised in Table 1. Blood film was unremarkable. Chest radiograph showed bilateral patchy consolidations in all lung fields (Figure 1). The patient was rapidly transferred to the intensive therapy unit (ITU) for intubation and lung-protective ventilation with regular ‘proning’.

**Table 1:** Blood tests results PT: prothrombin time; APTT: activated partial thromboplastin time; eGFR: estimated glomerular filtration rate.

Blood test	Value	Reference range
Haemoglobin	102	135-175 g/l
White cell count	10.8	4.0-11.0 X10^9^/l
Lymphocyte count	0.9	1.0-4.0 X10^9^/l
Platelets	122	135-450 X10^9^/l
PT	16.2	11-15 sec
APTT	28	26-37 sec
D-dimer	77,405	<500 ng/ml
Fibrinogen	1.13	2.0-4.5 g/l
Creatinine	83	59-104 µmol/l
Urea	12	2.5-7.8 mmol/l
eGFR	89	>90 ml/min/1.73 m^2^

A day into admission to ITU, it was noted that the patient had priapism with a dusky penis and a urinary catheter in situ. This prompted a Urology team consult. Penile aspiration was carried out in the ITU setting with the aspiration of 19 ml of dark hypoxic blood. Bilateral intra-cavernosal injection of a total of 1 mg of phenylephrine over 60 minutes was carried out, and detumescence was achieved. Penile blood gas analysis results are summarised in Table 2.

**Table 2:** Penile blood gas analysis results pCO_2_: partial pressure of carbon dioxide; pO_2_: partial pressure of oxygen; sO_2_: saturation of oxygen; TLBR: too low to be recorded; THBR: too high to be recorded.

Blood gas test	Value	Reference range
pH	TLBR	7.35-7.45
pCO_2_	THBR	4.7-6.4 kPa
pO_2_	TLBR	11.0-14.4 kPa
sO_2_	5.1	70%-80 %
Haemoglobin	185	135-175 g/l
Sodium	137	133-146 mmol/l
Potassium	9.3	3.5-5.3 mmol/l
Lactate	22	0.5-2.2 mmol/l

Sadly, later that night the patient became hypotensive with a substantial increase in noradrenaline requirements. Proning was carried out, and a referral was made to a tertiary centre for extra-corporeal membrane oxygenation; however, regrettably, the patient passed away. COVID-19 reverse transcription polymerase chain reaction (RT-PCR) endo-tracheal swabs returned positive.

## Discussion

The range of presentation of COVID-19 varies between a mild self-limiting upper respiratory tract illness to life-threatening severe hypoxia requiring ventilation in an ITU setting. The lung histological pattern in the post-mortem of patients who died from COVID-19 was primarily diffuse alveolar damage with peri-vascular T cell infiltration in the peripheral lung [2]. COVID-19 is unique amongst the causes of respiratory distress syndrome in that distinctive vascular features have been described, namely severe endothelial injury, widespread thrombosis and microangiopathy [3]. Excess microthrombi in alveolar capillaries and severe endothelialitis are the main differentiating histological features from the influenza virus or even other coronaviruses [2]. Vascular angiogenesis as a response to microscopic blood clots in the lungs of COVID-19 patients post-mortem was characteristic and a key differentiator between COVID-19 and other respiratory diseases.

A major hypothesis postulated for the development of extensive pulmonary microthrombi is via a reduction in angiotensin-converting enzyme 2 (ACE2) and the subsequent increase in oxygen-free radicals. This will lead to an increase in von Willebrand Factor (VWF) levels via disruption to the endothelial cell lining [4]. Numerous observational studies have described a link between COVID-19 infection and increased susceptibility to thrombosis via coagulopathy and disseminated intravascular coagulation (DIC) [5]. An association between survival with heparin use in COVID-19 infection in patients with coagulopathy due to sepsis and a raised D-dimer has been reported in previous pooled analyses [6].

Two clinicopathological subtypes of priapism exist, the high flow (non-ischaemic) and low flow (ischaemic). High-flow priapism involves the formation of arterio-cavernous shunts with the subsequent increased arterial flow, an intact veno-occlusive system and an elastic textured painless erection, with preservation of tissue oxygen supply and good erectile recovery in most cases [7]. This contrasts with low-flow priapism which involves tissue hypoxia and subsequent ischaemia, de-oxygenated blood pooling and venous stasis. Conditions predisposing to blood hyperviscosity, such as leukaemia and even haemodialysis, can also produce tissue hypoxia and fibrosis which leads to erectile tissue ischaemia and risk of permanent erectile dysfunction without appropriate intervention [8]. Ultrastructural examination in priapism reveals oedema and sinusoidal endothelial destruction with exposure of basement membrane with adherence of vascular platelets and vascular thrombi over a 24- to 48-hour period [9].

Priapism is considered a surgical emergency, especially the low-flow variety in which there is a greater complication rate and recovery of erectile function depends on prompt diagnosis and management [10]. The incidence of priapism is 1.5 per 100,000 with sickle cell disease being the commonest cause in children and medications being the commonest cause in adults [10]. Other causes of priapism include haematological disorders, metabolic conditions, trauma, haemodialysis, total parenteral nutrition, neurological disease and idiopathic priapism [10].

The aforementioned pathology underlying both types of priapism has been well described in the literature. Our understanding to date of COVID-19 and its vasculopathy has helped us hypothesise the reasons for low-flow priapism in our patient. The shared pathological hallmarks of COVID-19 infection and priapism, microemboli phenomena, arterial compromise, acute disseminated intravascular coagulation and tissue hypoxia, characterise both conditions, and this is the first reported case in the literature of such an association.

Vascular endothelial dysfunction in COVID-19 has been demonstrated in a recent study via direct viral infection causing endothelialitis and a dysregulated host inflammatory response [11]. The vasoconstriction resulting from this endothelial cell dysfunction is what subsequently leads to a pro-coagulant state, tissue oedema and organ ischaemia [12]. It is now felt there is a strong vascular thrombotic component to COVID-19 infection. Vascular co-morbidities such as hypertension, as our patient suffered from, have been shown to predispose patients to widespread endothelial cell dysfunction and subsequent end-organ ischaemia [13].

The role of endothelial nitric oxide synthase (eNOS) in endothelial cell dysfunction associated with COVID-19 and in low-flow priapism lends further support to both conditions sharing a common final pathway in distal vascular networks. Suppressed eNOS due to endothelial cell dysfunction and subsequent nitric oxide (NO) deficiency is a major contributory factor to vascular insufficiency and thrombus formation [14]. Loss of eNOS and subsequent reduction in NO availability due to endothelium damage in low-flow priapism have been widely described [15]. The chronic state of haemolysis and subsequent decreased NO availability, as seen in sickle cell disease, are the main pathologies underlying low-flow priapism and decreased endothelial NO [16].

The interaction between coronavirus viral S-protein and its receptor ACE2 has been shown to be affected by levels of NO and is a potential antiviral target [17]. It is therefore thought that restoring levels of NO can counteract endothelial cell dysfunction and subsequent vasoconstriction and thrombosis [18]. The continued use of ACE inhibitors or statins in patients with vascular disease who develop COVID-19 has been shown to confer a survival advantage in some centres, but introducing them as COVID-19-specific anti-thrombotic agents has yet to be studied [19]. Proposals for their potential benefits involve the role of ACE2 in mitigating the renin-angiotensin system (RAS) and reducing the release of pro-inflammatory cytokines. Increased activity of ACE2 increases the levels of angiotensin 1-7 (Ang1-7) and the activity of the angiotensin/Mas (Ang-Mas) receptor with subsequent anti-fibrotic and anti-inflammatory effects [20].

## Conclusions

Our case highlights the importance of vigilance for extra-pulmonary manifestations of COVID-19. We feel that the pro-thrombotic phenomena exhibited by COVID-19 infection affect distal vasculature in the same manner as low-flow priapism and can explain why our patient suffered from this particular urological complication. Low-flow priapism may be one of the many extra-pulmonary manifestations of COVID-19 secondary to the pro-thrombotic state associated with SARS-COV-2 infection.
